# Supplementary material for: Droplet microfluidics for time-resolved serial crystallography
Source: IUCrJ. 2024 Mar 1;11(Pt 2):237–48. doi: 10.1107/S2052252524001799 (PMC10916287; doi:10.1107/S2052252524001799)
Supplement: Supplementary file 5 [file m-11-00237-sup5.pdf]

IUCrJ

Volume 11 (2024)

Supporting information for article:

Droplet microfluidics for time-resolved serial crystallography

Jack Stubbs, Theo Hornsey, Niall Hanrahan, Luis Blay Esteban, Rachel Bolton, Martin Malý, Shibom Basu, Julien Orlans, Daniele de Sanctis, Jung-uk Shim, Patrick D. Shaw Stewart, Allen M. Orville, Ivo Tews and Jonathan West

Table S1 Droplet microfluidic conditions for preparing lysozyme crystals.

Junction Dimensions (*w*, *h*) (see Fig. 1A)	QX200 Fluoro-oil	Mother Liquor	Lysozyme	Droplet Diameter	Droplet Volume	Droplet Frequency
125x100 μm	60 μL/min	16 μL/min	4 μL/min	112.9 μm	754 pL	0.44 kHz
70x75 μm	37.5 μL/min	10 μL/min	2.5 μL/min	71.8 μm	194 pL	1.07 kHz
50x50 μm	22.5 μL/min	6 μL/min	1.5 μL/min	54.4 μm	84.3 pL	1.48 kHz
35x35 μm	15 μL/min	3 μL/min	0.75 μL/min	33.5 μm	19.7 pL	3.18 kHz
22x20 μm	8 μL/min	2 μL/min	0.5 μL/min	23.2 μm	6.54 pL	6.37 kHz
20x12 μm	8 μL/min	1.6 μL/min	0.4 μL/min	17.6 μm	2.85 pL	11.7 kHz
10x12 μm	5 μL/min	1 μL/min	0.25 μL/min	11.9 μm	0.89 pL	23.3 kHz

Table S2 Droplet microfluidic conditions for preparing Pdx1 crystals.

Junction Dimensions (*w*, *h*)	QX200 Fluoro-oil	Mother Liquor + Seed	Pdx1	Droplet Diameter	Droplet Volume	Droplet Frequency
70x75 μm	90 μL/min	26.67 μL/min	13.33 μL/min	74.8 μm	219 pL	3.04 kHz
50x50 μm	60 μL/min	13.33 μL/min	6.67 μL/min	48.6 μm	60.1 pL	5.54 kHz
35x35 μm	40 μL/min	6.67 μL/min	3.33 μL/min	32.5 μm	18.0 pL	9.27 kHz
20x12 μm	16 μL/min	2.67 μL/min	1.33 μL/min	19.6 μm	3.94 pL	16.9 kHz
10x12 μm	8 μL/min	1.33 μL/min	0.67 μL/min	12.7 μm	1.07 pL	31.1 kHz
10x5 μm	2x1 μL/min	0.2 μL/min	0.1 μL/min	5.38 μm	82 fL	61.3 kHz

Table S3 Droplet microfluidic conditions for preparing trypsin type I needle crystals.

Junction Dimensions (*w*, *h*)	QX200 Fluoro-oil	Mother Liquor	Trypsin	Seeds	Droplet Diameter	Droplet Volume	Droplet Frequency
70x75 μm	54 μL/min	6 μL/min	6 μL/min	6 μL/min	75 μm	221 pL	1.36 kHz
50x50 μm	27 μL/min	3 μL/min	3 μL/min	3 μL/min	50 μm	65 pL	2.29 kHz
35x35 μm	9 μL/min	1 μL/min	1 μL/min	1 μL/min	35 μm	22 pL	2.22 kHz

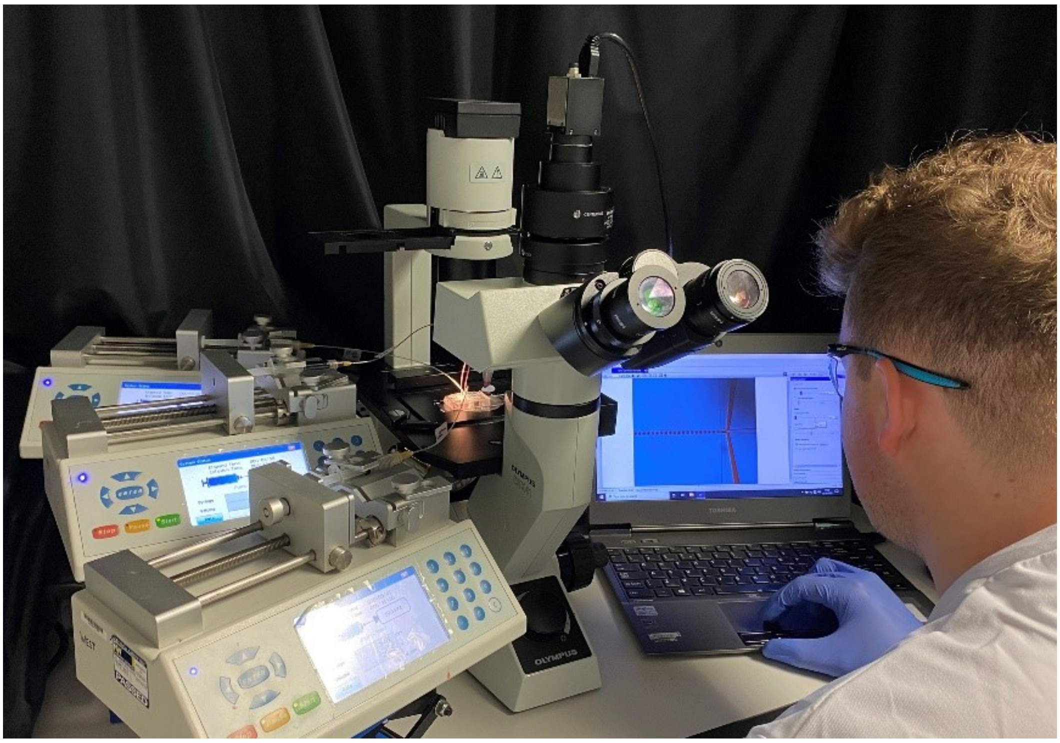

Figure S1 Droplet microfluidics experimental setup.

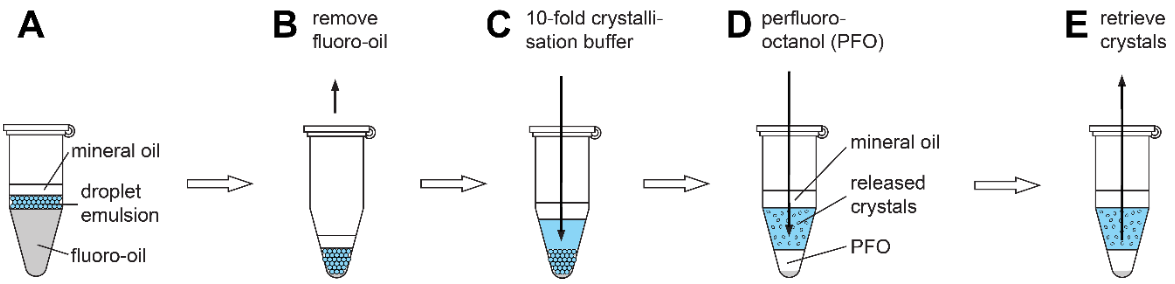

Figure S2 *Breaking the emulsion*. Droplets are collected under a mineral oil overlay, with the buoyant nature causing an emulsion to form above the fluoro-oil carrier (A). The fluoro-oil is removed (B), and a ~10-fold volume of crystallisation buffer relative to emulsion volume is added to the emulsion (C). Next, a 1-fold volume of perfluoro-1-octanol (PFO) is added, and gently mixed to coalesce all droplets into a single aqueous volume (D). This volume and the crystals within it are retrieved for analysis (E).

Serial Synchrotron Crystallography (SSX) Data Processing

Diffraction patterns were processed using CrystFEL (v.0.10.2; White *et al.,* 2012, 2019). Images were stored in a hdf5 stream and initial hit finding was performed with Cheetah (Barty *et al.,* 2014). Detector geometry refinement was performed with *geoptimiser* (Yefanov *et al.,* 2015) and a smaller subset of 1000 images for each dataset, ensuring high indexing rates. Hits were indexed with x*gandalf* (Gevorkov *et al.,* 2019) and integrated using the following parameters: `--peaks=peakfinder8 --multi --int-radius=4.0,6.0,10.0 --tolerance=5,5,5,1.5,1.5,1.5 --peak-radius=4,6,10 --min-peaks=30 --min-snr=4.0 --threshold=1500 --local-bg-radius=5 --min-res=80 --max-res=1200 --min-pix-count=3 --max-pix-count=200`. Indexing ambiguities present in the Pdx1 datasets (arising from the *H*3 space group) were resolved using *ambigator* and the following parameters: `-y 3_H --operator=k,h,-l --iterations=20 --highres=3.0`. Data were merged using *partialator* with partialities, post-refinement and scaling using the following parameters: `-y 4/mmm` (Lysozyme) or `-y 3_H` (Pdx1) `--model=xsphere --iterations=1`. Figures of merit (Figure S3) and .mtz files for crystallographic structure determination were generated using the import_serial task, currently available in the latest version of CCP4 (8.0.016) (Agirre *et al.,* 2023) (https://github.com/MartinMalyMM/import_serial).

Lysozyme control preparations produced 29,954 indexable patterns, resulting in 5,990 indexed patterns/μL, and droplet preparations produced 22,304 indexable patterns, resulting in 4,460 indexed patterns/μL. In comparison, Pdx1 control preparations produced 19,325 indexable patterns, resulting in 1,288 indexed patterns/μL, and droplet preparations produced 20,635 indexable patterns respectively, resulting in 1,375 indexed patterns/μL. The number of integrated lattices is much greater than indexed images, especially in the case of lysozyme where multiple lattices were observed in the same pattern.

Structure solution and refinement

Molecular replacement with MOLREP (Vagin & Teplyakov, 2010) used 6H79 (Monteiro *et al.*, 2019) and 7NHF (Rodrigues *et al.*, 2022) as search models. Iterative rounds of model building and refinement used Coot (Emsley *et al.*, 2010) and REFMAC5 (Murshudov *et al.*, 2011) within CCP4i2 (Potterton *et al.*, 2018). Optimised refinement parameters were set using the PDB-REDO server (Joosten *et al.*, 2014). Model validation was performed using the wwPDB validation service (Berman *et al.*, 2003) and MolProbity (Williams *et al.*, 2018) prior to deposition. Full data collection and refinement statistics are shown in Table 1. Structure factor files and atomic coordinates have been deposited in the PDB with accession codes 8S2U (Lysozyme Control), 8S2V (Lysozyme Droplet), 8S2W (Pdx1 Control) and 8S2X (Pdx1 Droplet).

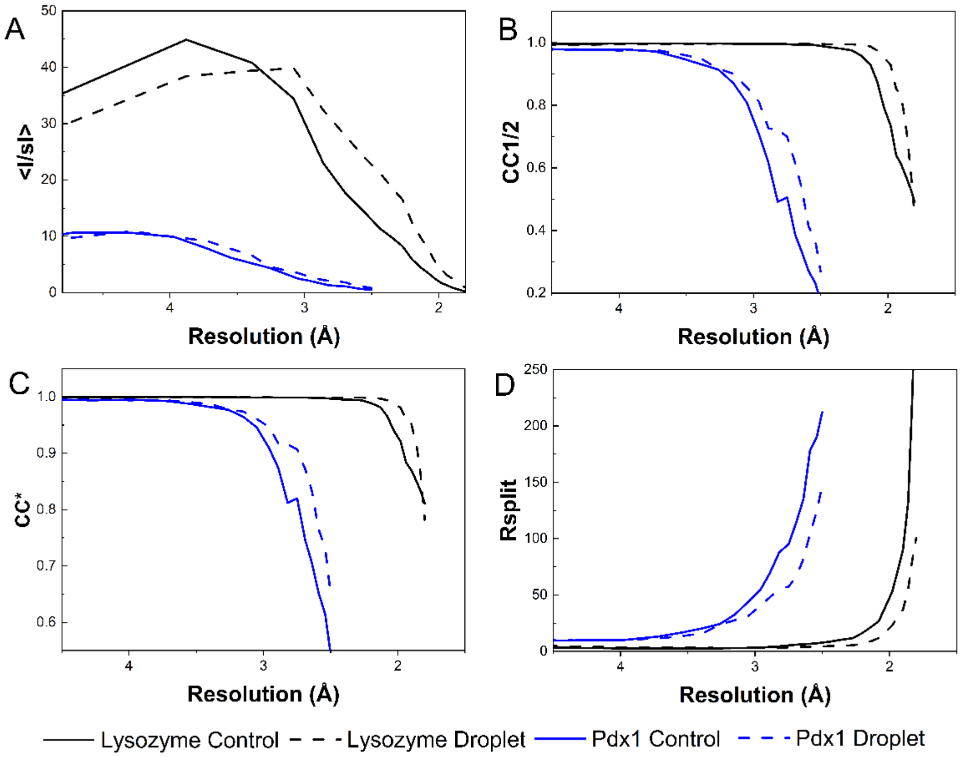

Figure S3 Plots of figures of merit. (A) Mean signal-to-noise ratio ⟨I/σ(I)⟩, (B) Pearson's correlation coefficient $CC_{1/2}$, (C) CC^* and (D) R_{split} against resolution comparing lysozyme and Pdx1 crystals grown in batch with those grown in droplets.

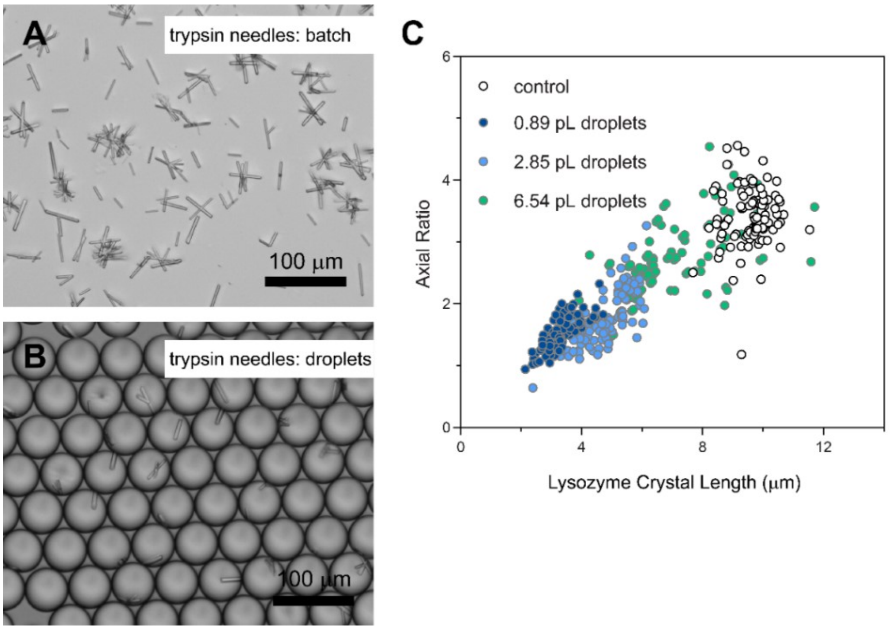

Figure S4 Trypsin needles and confinement effects on lysozyme axial ratio. (A) Trypsin needles prepared in batch have various lengths with an axial ratio of ~10, (B) whereas crystallisation in droplets produces an axial ratio of ~5. (C) The axial ratio of lysozyme crystals grown in droplets tends to unity as droplets are miniaturised below 1 pL.

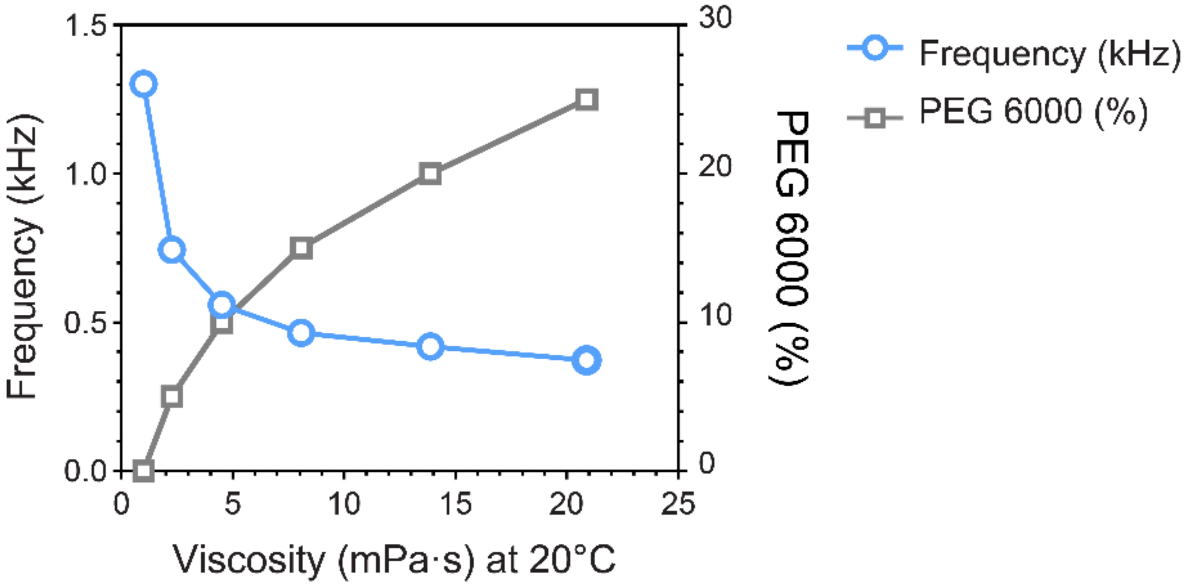

Figure S5 Viscosity effect on droplet generation throughput. Droplet microfluidics can manage high viscosity crystallisation buffers, although increased viscosity decreases the droplet generation frequency.

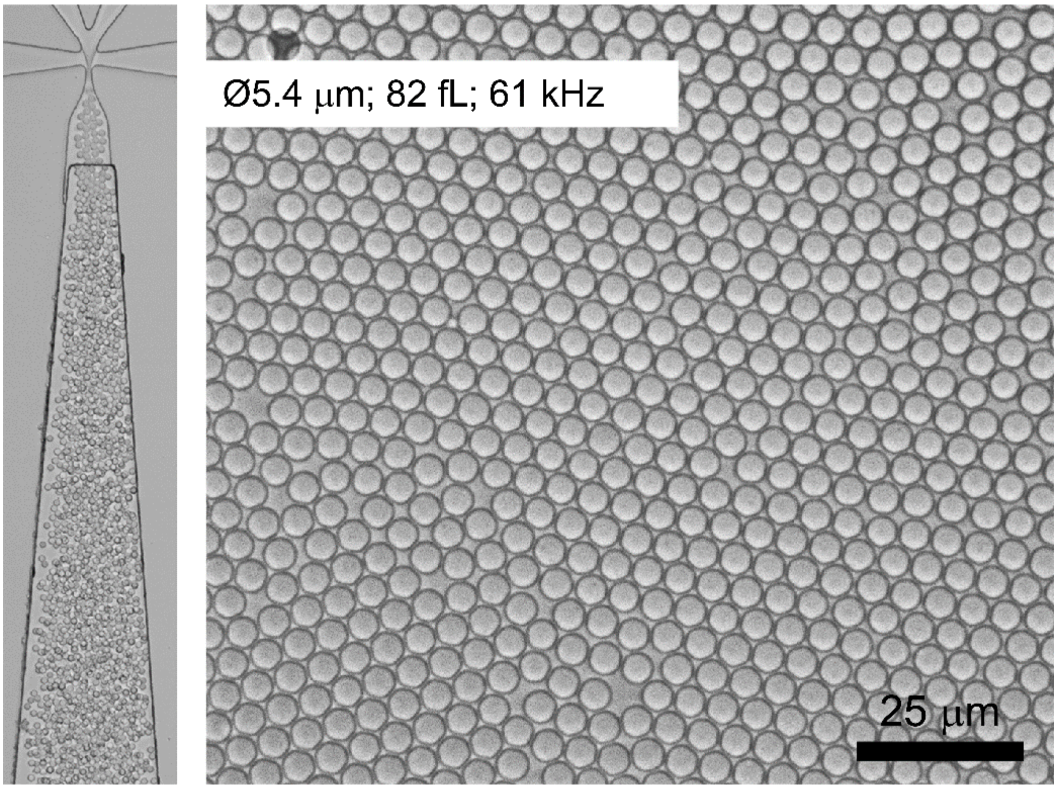

Figure S6 Femtolitre droplets for Pdx1 crystallisation. Miniaturisation enables the high throughput (61 kHz) generation of ~5-µm-diameter, 82 fL droplets (left). Even with seeding, Pdx1 crystals were not apparent with a 60x/1.4NA oil immersion objective (right).

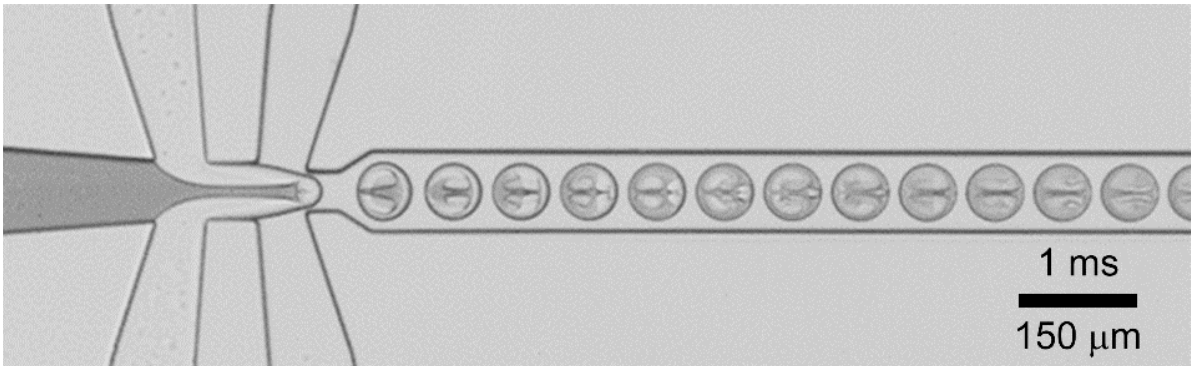

Figure S7 Circulations within droplets provide convection to drive rapid micromixing.

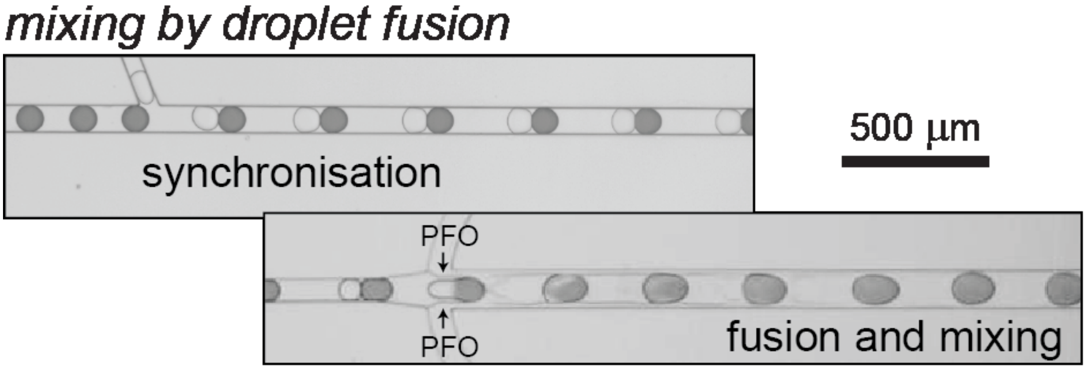

Figure S8 Droplet synchronisation and 1:1 coupling followed by surfactant exchange using PFO to trigger droplet fusion and mixing (Video S2 and S3). The synchronisation channel is 75x40 µm (*w*,*h*) with a droplet velocity of 120 mm/s. The droplet fuse and mix channel is 125x40 µm (w,h) with a droplet velocity of 85 mm/s. 200 pL crystal droplets are fused with 225 pL dye droplets with mixing achieved in ~7 milliseconds.

Supporting references

Agirre, J., Atanasova, M., Bagdonas, H., Ballard, C. B., Baslé, A., Beilsten-Edmands, J., Borges, R. J., Brown, D. G., Burgos-Mármol, J. J., Berrisford, J. M., Bond, P. S., Caballero, I., Catapano, L., Chojnowski, G., Cook, A. G., Cowtan, K. D., Croll, T. I., Debreczeni, J. É., Devenish, N. E., Dodson, E. J., Drevon, T. R., Emsley, P., Evans, G., Evans, P. R., Fando, M., Foadi, J., Fuentes-Montero, L., Garman, E. F., Gerstel, M., Gildea, R. J., Hatti, K., Hekkelman, M. L., Heuser, P., Hoh, S. W., Hough, M. A., Jenkins, H. T., Jiménez, E., Joosten, R. P., Keegan, R. M., Keep, N., Krissinel, E. B., Kolenko, P., Kovalevskiy, O., Lamzin, V. S., Lawson, D. M., Lebedev, A. A., Leslie, A. G. W., Lohkamp, B., Long, F., Malý, M., McCoy, A. J., McNicholas, S. J., Medina, A., Millán, C., Murray, J. W., Murshudov, G. N., Nicholls, R. A., Noble, M. E. M., Oeffner, R., Pannu, N. S., Parkhurst, J. M., Pearce, N., Pereira, J., Perrakis, A., Powell, H. R., Read, R. J., Rigden, D. J., Rochira, W., Sammito, M., Sánchez Rodríguez, F., Sheldrick, G. M., Shelley, K. L., Simkovic, F., Simpkin, A. J., Skubak, P., Sobolev, E., Steiner, R. A., Stevenson, K., Tews, I., Thomas, J. M. H., Thorn, A., Valls, J. T., Uski, V., Usón, I., Vagin, A., Velankar, S., Vollmar, M., Walden, H., Waterman, D., Wilson, K. S., Winn, M. D., Winter, G., Wojdyr, M. & Yamashita, K. (2023). Acta Cryst D 79, 449–461.

Barty, A., Kirian, R.A., Maia, F.R., Hantke, M., Yoon, C.H., White, T.A. and Chapman, H. (2014). J Appl Cryst, 47(3), pp.1118-1131.

Berman, H., Henrick, K. & Nakamura, H. (2003). Nat Struct Mol Biol 10, 980–980.

Emsley, P., Lohkamp, B., Scott, W. G. & Cowtan, K. (2010). Acta Cryst D 66, 486–501.

Gevorkov, Y., Yefanov, O., Barty, A., White, T.A., Mariani, V., Brehm, W., Tolstikova, A., Grigat, R.R. and Chapman, H.N. (2019). Acta Cryst A 75(5), pp.694-704.

Joosten, R. P., Long, F., Murshudov, G. N. & Perrakis, A. (2014). *IUCrJ* **1**, 213–220.

Monteiro, D. C. F., Vakili, M., Harich, J., Sztucki, M., Meier, S. M., Horrell, S., Josts, I. & Trebbin, M. (2019). J Synchrotron Rad 26, 406–412.

Murshudov, G. N., Skubák, P., Lebedev, A. A., Pannu, N. S., Steiner, R. A., Nicholls, R. A., Winn, M. D., Long, F. & Vagin, A. A. (2011). Acta Cryst D 67, 355–367.

Potterton, L., Agirre, J., Ballard, C., Cowtan, K., Dodson, E., Evans, P. R., Jenkins, H. T., Keegan, R., Krissinel, E., Stevenson, K., Lebedev, A., McNicholas, S. J., Nicholls, R. A., Noble, M., Pannu, N. S., Roth, C., Sheldrick, G., Skubak, P., Turkenburg, J., Uski, V., von Delft, F., Waterman, D., Wilson, K., Winn, M. & Wojdyr, M. (2018). Acta Cryst D 74, 68–84.

Rodrigues, M. J., Giri, N., Royant, A., Zhang, Y., Bolton, R., Evans, G., Ealick, S. E., Begley, T. & Tews, I. (2022). *RSC Chem. Biol.* **3**, 227–230.

Vagin, A. & Teplyakov, A. (2010). Acta Cryst D 66, 22–25.

White, T.A., (2019). Acta Cryst D 75(2), pp.219-233.

White, T.A., Kirian, R.A., Martin, A.V., Aquila, A., Nass, K., Barty, A. and Chapman, H.N. (2012). J Appl Cryst, 45(2), pp.335-341.

Williams, C. J., Headd, J. J., Moriarty, N. W., Prisant, M. G., Videau, L. L., Deis, L. N., Verma, V., Keedy, D. A., Hintze, B. J., Chen, V. B., Jain, S., Lewis, S. M., Arendall III, W. B., Snoeyink, J., Adams, P. D., Lovell, S. C., Richardson, J. S. & Richardson, D. C. (2018). *Protein Science* **27**, 293–315.

Yefanov, O., Mariani, V., Gati, C., White, T.A., Chapman, H.N. and Barty, A., (2015). Optics Express, 23(22), pp.28459-28470.
